# Supplementary material for: Diagnostic Accuracy of Touchscreen-Based Tests for Mild Cognitive Disorders: A Systematic Review and Meta-Analysis
Source: Diagnostics (Basel). 2025 Sep 18;15(18):2383. doi: 10.3390/diagnostics15182383 (PMC12468971; doi:10.3390/diagnostics15182383)
Supplement: Supplementary file 1 [file diagnostics-15-02383-s001.zip › Supplementary_material.pdf]

Supplementary material

Title: Diagnosis accuracy of touchscreen-based testings for mild cognitive disorders: a systematic review and meta-analysis

Nathavy Um Din[1], Florian Maronnat[2], Bruno Oquendo[3], Sylvie Pariel[3], Carmelo Lafuente-Lafuente[3,4], Fadi Badra[1] and Joël Belmin[1,3] *

[1] Sorbonne Université, Université Sorbonne Paris-Nord, Laboratoire d'Informatique Médicale et d'Ingénierie des Connaissances en e-Santé, LIMICS, France ; nathavy.umdin@gmail.com

[2] Université de Bretagne Occidentale, Centre hospitalo-universitaire de Brest, 29200 Brest, France ; florian.maronnat@chu-brest.fr

[3] Hôpital Charles Foix, 7 avenue de la République, Ivry sur Seine, Sorbonne Université, 91-105 boulevard de l'Hôpital, 75013 Paris, France; j.belmin@aphp.fr

[4] Clinical Epidemiology and Ageing (CEpiA) Team, Université Paris Est-Créteil, INSERM, IRMB, F-9400 Créteil, France ; carmelo.lafuente@aphp.fr

* Correspondence: j.belmin@aphp.fr

PRISMA-DTA Checklist

Section/topic	#	PRISMA-DTA Checklist Item	Reported on page #
**TITLE / ABSTRACT**			
Title	1	Identify the report as a systematic review (+/- meta-analysis) of diagnostic test accuracy (DTA) studies.	1
Abstract	2	Abstract: See PRISMA-DTA for abstracts.	1
**INTRODUCTION**			
Rationale	3	Describe the rationale for the review in the context of what is already known.	2
Clinical role of index test	D1	State the scientific and clinical background, including the intended use and clinical role of the index test, and if applicable, the rationale for minimally acceptable test accuracy (or minimum difference in accuracy for comparative design).	2
Objectives	4	Provide an explicit statement of question(s) being addressed in terms of participants, index test(s), and target condition(s).	2
**METHODS**			
Protocol and registration	5	Indicate if a review protocol exists, if and where it can be accessed (e.g., Web address), and, if available, provide registration information including registration number.	1-3
Eligibility criteria	6	Specify study characteristics (participants, setting, index test(s), reference standard(s), target condition(s), and study design) and report characteristics (e.g., years considered, language, publication status) used as criteria for eligibility, giving rationale.	3-4
Information sources	7	Describe all information sources (e.g., databases with dates of coverage, contact with study authors to identify additional studies) in the search and date last searched.	3-4
Search	8	Present full search strategies for all electronic databases and other sources searched, including any limits used, such that they could be repeated.	3 and Table A1 (Appendices)
Study selection	9	State the process for selecting studies (i.e., screening, eligibility, included in systematic review, and, if applicable, included in the meta-analysis).	3-5
Data collection process	10	Describe method of data extraction from reports (e.g., piloted forms, independently, in duplicate) and any processes for obtaining and confirming data from investigators.	3-4
Definitions for data extraction	11	Provide definitions used in data extraction and classifications of target condition(s), index test(s), reference standard(s) and other characteristics (e.g. study design, clinical setting).	3-5 and Table A1 of Appendices
Risk of bias and applicability	12	Describe methods used for assessing risk of bias in individual studies and concerns regarding the applicability to the review question.	4
Diagnostic accuracy measures	13	State the principal diagnostic accuracy measure(s) reported (e.g. sensitivity, specificity) and state the unit of assessment (e.g. per-patient, per-lesion).	4
Synthesis of results	14	Describe methods of handling data, combining results of studies and describing variability between studies. This could include, but is not limited to: a) handling of multiple definitions of target condition. b) handling of multiple thresholds of test positivity, c) handling multiple index test readers, d) handling of indeterminate test results, e) grouping and comparing tests, f) handling of different reference standards	4

PRISMA-DTA Checklist

Section/topic	#	PRISMA-DTA Checklist Item	Reported on page #
Meta-analysis	D2	Report the statistical methods used for meta-analyses, if performed.	4
Additional analyses	16	Describe methods of additional analyses (e.g., sensitivity or subgroup analyses, meta-regression), if done, indicating which were pre-specified.	4
**RESULTS**			
Study selection	17	Provide numbers of studies screened, assessed for eligibility, included in the review (and included in meta-analysis, if applicable) with reasons for exclusions at each stage, ideally with a flow diagram.	4-5
Study characteristics	18	For each included study provide citations and present key characteristics including: a) participant characteristics (presentation, prior testing), b) clinical setting, c) study design, d) target condition definition, e) index test, f) reference standard, g) sample size, h) funding sources	6 + Tables A2 and A3
Risk of bias and applicability	19	Present evaluation of risk of bias and concerns regarding applicability for each study.	6-7 + Table A4
Results of individual studies	20	For each analysis in each study (e.g. unique combination of index test, reference standard, and positivity threshold) report 2x2 data (TP, FP, FN, TN) with estimates of diagnostic accuracy and confidence intervals, ideally with a forest or receiver operator characteristic (ROC) plot.	7-9, 22-23, "Data available"
Synthesis of results	21	Describe test accuracy, including variability; if meta-analysis was done, include results and confidence intervals.	7-9
Additional analysis	23	Give results of additional analyses, if done (e.g., sensitivity or subgroup analyses, meta-regression; analysis of index test: failure rates, proportion of inconclusive results, adverse events).	7-9, 22-23
**DISCUSSION**			
Summary of evidence	24	Summarize the main findings including the strength of evidence.	9-10
Limitations	25	Discuss limitations from included studies (e.g. risk of bias and concerns regarding applicability) and from the review process (e.g. incomplete retrieval of identified research).	10
Conclusions	26	Provide a general interpretation of the results in the context of other evidence. Discuss implications for future research and clinical practice (e.g. the intended use and clinical role of the index test).	11
**FUNDING**			
Funding	27	For the systematic review, describe the sources of funding and other support and the role of the funders.	11

Adapted From: McInnes MDF, Moher D, Thombs BD, McGrath TA, Bossuyt PM, The PRISMA-DTA Group (2018). Preferred Reporting Items for a Systematic Review and Meta-analysis of Diagnostic Test Accuracy Studies: The PRISMA-DTA Statement. JAMA. 2018 Jan 23;319(4):388-396. doi: 10.1001/jama.2017.19163.

PRISMA-DTA Checklist

Section/topic	#	PRISMA-DTA for Abstracts Checklist item	Reported on page #
**TITLE and PURPOSE**			
Title	1	Identify the report as a systematic review (+/- meta-analysis) of diagnostic test accuracy (DTA) studies.	1
Objectives	2	Indicate the research question, including components such as participants, index test, and target conditions.	1
**METHODS**			
Eligibility criteria	3	Include study characteristics used as criteria for eligibility.	1
Information sources	4	List the key databases searched and the search dates.	1
Risk of bias & applicability	5	Indicate the methods of assessing risk of bias and applicability.	1
Synthesis of results	A1	Indicate the methods for the data synthesis.	1
**RESULTS**			
Included studies	6	Indicate the number and type of included studies and the participants and relevant characteristics of the studies (including the reference standard).	1
Synthesis of results	7	Include the results for the analysis of diagnostic accuracy, preferably indicating the number of studies and participants. Describe test accuracy including variability; if meta-analysis was done, include summary results and confidence intervals.	1
**DISCUSSION**			
Strengths and limitations	9	Provide a brief summary of the strengths and limitations of the evidence	1
Interpretation	10	Provide a general interpretation of the results and the important implications.	1
**OTHER**			
Funding	11	Indicate the primary source of funding for the review.	1
Registration	12	Provide the registration number and the registry name	1

Adapted From: McInnes MDF, Moher D, Thombs BD, McGrath TA, Bossuyt PM, The PRISMA-DTA Group (2018). Preferred Reporting Items for a Systematic Review and Meta-analysis of Diagnostic Test Accuracy Studies: The PRISMA-DTA Statement. JAMA. 2018 Jan 23;319(4):388-396. doi: 10.1001/jama.2017.19163.
